# Increased knee torsional misalignment associated with femoral torsion is related to non-contact anterior cruciate ligament injury: a case–control study

**DOI:** 10.1186/s13018-024-04609-y

**Published:** 2024-02-06

**Authors:** Dehua Wang, Hengkai Fan, Linlin Hu, Xi Liang, Wei Huang, Ke Li

**Affiliations:** 1 Department of Orthopedics, The First Affiliated Hospital of Chongqing Medical University, 1 Youyi Road, Yuanjiagang Yuzhong District, Chongqing, 400016 China; 2 Orthopedic Laboratory of Chongqing Medical University, Chongqing, China; 3 Department of Orthopaedics, Lanzhou University Second Hospital, Orthopaedics Key Laboratory of Gansu Province, Gansu, China; 4 Department of Radiology, The First Affiliated Hospital of Chongqing Medical University, Chongqing, China

**Keywords:** ACL injury, Risk factors, Knee torsion, Distal femoral torsion, Posterior femoral condylar torsion, Proximal tibial torsion

## Abstract

**Background:**

Altered axial biomechanics of the knee are recognized as a risk factor for non-contact anterior cruciate ligament (ACL) injury. However, the relationship of knee and segmental torsion to non-contact ACL and combined anterolateral ligament (ALL) injury is unclear. This study aims to determine the relationship of knee and segmental torsion to non-contact ACL injury and to explore their relationship with ALL injuries.

**Methods:**

We divided 122 patients with arthroscopically confirmed non-contact ACL injuries into an ACL injury group (isolated ACL injury, 63 patients) and an ACL + ALL injury group (ACL combined with ALL injury,59 patients). Additionally, 90 normal patients with similar age, gender and body mass index (BMI) were matched as a control group. The tibial tubercle-trochlear groove (TT-TG) distance, distal femoral torsion (DFT), posterior femoral condylar torsion (PFCT) and proximal tibial torsion (PTT) were measured using magnetic resonance imaging (MRI). We assessed the differences between the groups using an independent samples t test and utilized receiver operating characteristic (ROC) curves to determine the cut-off value for the increased risk of ACL injury.

**Results:**

In patients with ACL injury, the measurements of the TT-TG (11.8 ± 3.1 mm), DFT (7.7° ± 3.5°) and PFCT (3.6° ± 1.3°) were significantly higher compared to the control group (9.1 ± 2.4 mm, 6.3° ± 2.7° and 2.8° ± 1.3°, respectively; *P* < 0.05), but the PTT did not differ between the two groups. The TT-TG, DFT and PFCT were not significantly larger in patients combined with ALL injury. ROC curve analysis revealed ACL injury is associated with TT-TG, DFT and PFCT.

**Conclusions:**

Knee torsional alignment is associated with ACL injury, predominantly in the distal femur rather than the proximal tibia. However, its correlation with ALL injury remains unclear. These findings may help identify patients at high risk for non-contact ACL injury and inform the development of targeted prevention and treatment strategies.

## Introduction

Non-contact anterior cruciate ligament (ACL) injury accounts for more than half of all ACL injuries [1]. This type of injury primarily results by the mechanism of pivot shift, whereby ACL injury occurs when the tibia is valgus and internally rotated relative to the femur during knee motion resulting in greater stresses on the ACL [2]. To prevent non-contact ACL injury, several studies have investigated possible risk factors such as body mass index (BMI), neuromuscular defects, hormone levels, joint laxity and sex [3–7]. Moreover, several morphological characteristics of the bones can affect the biomechanics of the knee and increase the risk of ACL injury [8–11].

In recent years, many studies have demonstrated that altered biomechanics in the transverse plane is the core of the mechanism of ACL injury, and abnormal torsion changes the rotational stresses and increases the risk of ACL injury [12–14]. Alpay et al. identified femoral anteversion leading to abnormal torsion of the infratrochanteric femur as a risk factor for ACL injury [14]. The tibial tubercle to trochlear groove (TT-TG) distance, an indicator of knee torsion, is an essential factor in non-contact ACL injury, and a larger TT-TG increases tibial internal rotation, exposing the ACL to greater tension, thus increasing the risk of injury [15–17]. Non-contact ACL injury is usually due to a pivot-shift mechanism resulting from the combined action of the femur and tibia [2]. Therefore, distal femoral torsion (DFT), posterior femoral condylar torsion (PFCT), and proximal tibial torsion (PTT) may cause non-contact ACL injuries.

The anterolateral ligament (ALL) plays an essential role in the rotational stability of the knee, and its injury results in greater rotational stress on the knee, which is usually accompanied by a more severe knee injury [18, 19]. However, whether knee torsion is correlated with ACL combined ALL injuries is unclear. Therefore, understanding the torsion of various knee components may provide new insights into the mechanism of non-contact ACL injury.

The objective of this study is to measure the TT-TG, DFT, PFCT and PTT using magnetic resonance imaging (MRI) to explore the association between knee torsion and non-contact ACL injury using segmental analysis and to explore their relationship with combined ALL injury.

## Material and methods

This study was approved by the Ethics Committee of The First Affiliated Hospital of Chongqing Medical University (IRB, NO. 2019-345). Retrospective data were collected from 603 patients diagnosed with ACL injury at our centre between 2015 and 2022. The requirement for informed consent was waived because this was a retrospective imaging study.

### Patient selection

**Fig. 1:**
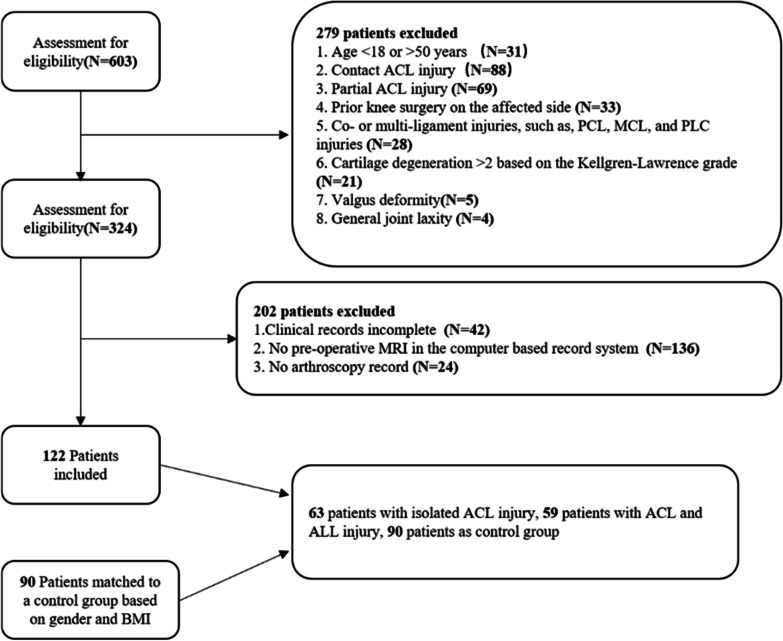
The flowchart of patient selection

After the screening, 122 patients with non-contact ACL injury were included in the experimental group, which was divided into the ACL injury group comprising patients with isolated ACL injury (41 men, 22 women) and the ACL + ALL injury group consisting of patients with ACL + ALL injury (40 men and 19 women), based on the presence of combined ALL injury. Furthermore, 90 normal patients (60 men, 30 women), who had undergone knee MRI at our hospital, were included as the control group matched by sex and BMI. The inclusion criteria were:1. patients aged 18–50 years; 2. availability of complete preoperative radiographs, MRI and medical records; 3. ACL injuries were defined based on arthroscopy. The exclusion criteria were:1. patients with contact ACL injury; 2. patients with partial ACL injuries; 3. surgical history of the injured knee; 4. patients with joint or multiple ligament injuries, such as the posterior cruciate ligament, medial and lateral collateral ligament, and other injuries; 5. patients with Kellgren–Lawrence grades > 2; 6. patients with obvious valgus deformities; 7. general joint laxity. A detailed flowchart illustrating patient selection is presented in Fig. 1.

### Data collection

**Fig. 2:**
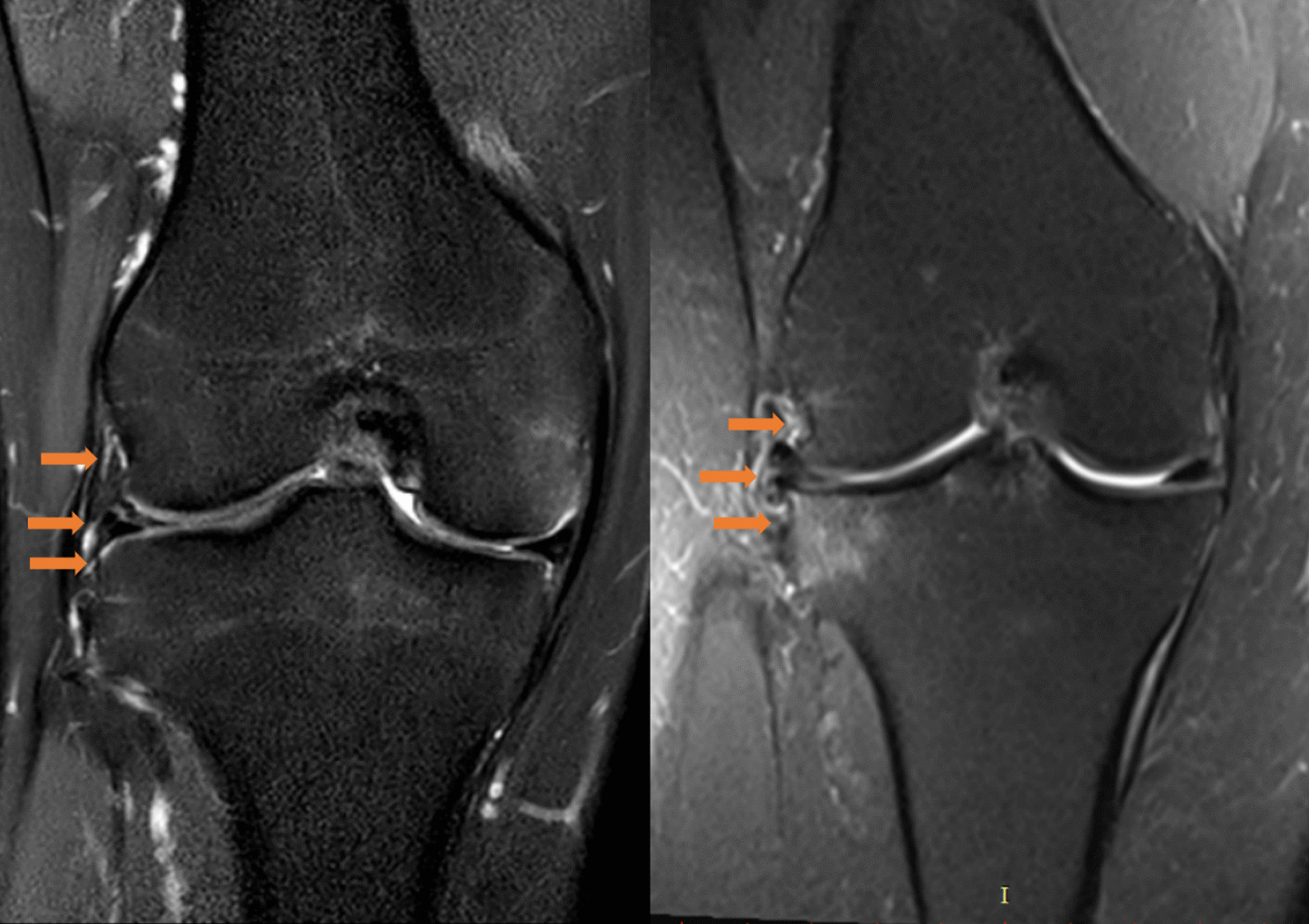
Representative magnetic resonance images of normal and abnormal ALL. ALL, anterolateral ligament

Patients' demographics were reviewed from the medical reports. Coronal, sagittal and axial images of MRI were independently evaluated by two orthopaedic surgeons. For ALL status assessment, T2-weighted coronal plane images were utilized. ALL injury was defined as discontinuity of ALL fibres or with periligamentous oedema, or proximal or distal detachment, with or without Segond fracture [18, 20] (Fig. 2).

**Fig. 3:**
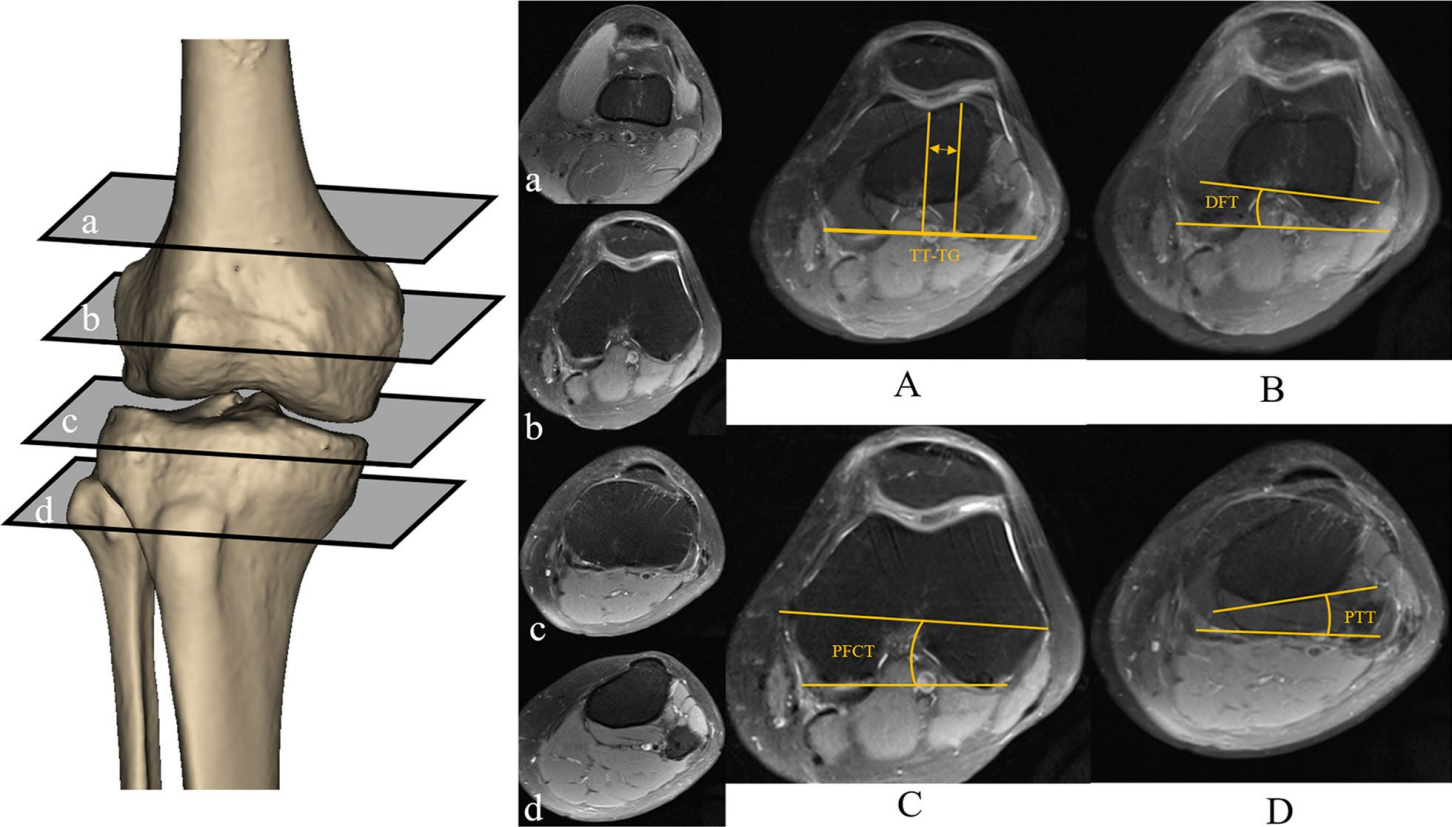
Measurements of TT-TG, DFT, PFCT and PTT. a, Slice above the gastrocnemius insertion of the distal femur. b, Slice of the gastrocnemius insertion of the distal femur. c, Slice of the proximal tibia. d, Slice of the level of the tibial tuberosity. **A** The TT-TG distance is the superposition of slices b and d. The TT-TG distance is defined as the distance measured on MRI between the most anterior part of the tibial trochanter and the middle of the trochlear groove. **B** The DFT is the superposition of slices a and b. The DFT is defined as the angle between the posterior cortical line of the distal femur and the posterior condylar line. **C** PFCT is defined as the angle between the line passing through the medial sulcus/apex of lateral epicondyle and the line passing through the posterior condylar axis on the slice b. **D** The PTT is the superposition of slices c and d. The PTT is defined as the angle between the line that joins the posterior border of the tibial plateaus at the level of the tibial insertion of the posterior cruciate ligament and the line that joins the posterior border of the tibia at the level of the tibial tubercle. TT-TG, tibial tubercle to trochlear groove. DFT, distal femoral torsion. PFCT, posterior femoral condylar torsion. PTT, proximal tibial torsion

All MRI data were downloaded, and all parameters were measured using Radiant DICOM Viewer (Medixant, Poland). TT-TG, DFT, PFCT and PTT measurements were based on previously published methods for axial MRI assessment. Specifically, the TT-TG distance was defined as the distance between the most anterior part of the tibial trochanter and the middle of the trochlear groove; the DFT was defined as the angle between the posterior cortical line above the gastrocnemius insertion of the distal femur and the posterior condylar line; the PFCT was defined as the angle between the line passing through the medial sulcus/apex of the lateral epicondyle and the line passing through the posterior condylar axis on the same slice and the PTT was defined as the angle between the line that joins the posterior border of the tibial plateaus at the level of the tibial insertion of the posterior cruciate ligament and the line that joins the posterior border of the tibia at the level of the tibial tubercle (Fig. 3) [16, 21–23]. The software provided measurements in mm and degrees with two decimal points. We eventually reported to one decimal point. Two sports medicine-specialised orthopaedic surgeons (DHW and HKF) measured all variables, and ultimately the mean of the two physicians' measurements was reported.

### Statistical analysis

Statistical analyses were performed with the SPSS software 23.0 (IBM, New York, USA). Measured data including age, BMI, TT-TG, DFT, PFCT and PTT of the study subjects were assessed by the Shapiro–Wilk test to conform to normal distribution and were shown as mean ± standard deviation, and the independent samples t test was used for comparisons between groups. Count data (sex and injury side) were described as rates, and the Chi-square test was used for comparisons between groups. The area under the curve (AUC) and 95% confidence intervals (CIs) for TT-TG, DFT, PFCT and PTT were plotted using receiver operating characteristic (ROC) curves, and using the Youden index, we determined the cut-off value with the greatest sensitivity and specificity. Subsequently, odds ratios (OR) were calculated to evaluate whether TT-TG, DFT, PFCT and PTT were risk factors for ACL injury. A statistically significant difference was defined as *P* < 0.05. With a power of 80%, α level of 0.05 and 95% confidence level, the number of subjects needed was calculated as 25 per group.

## Results

**Fig. 4:**
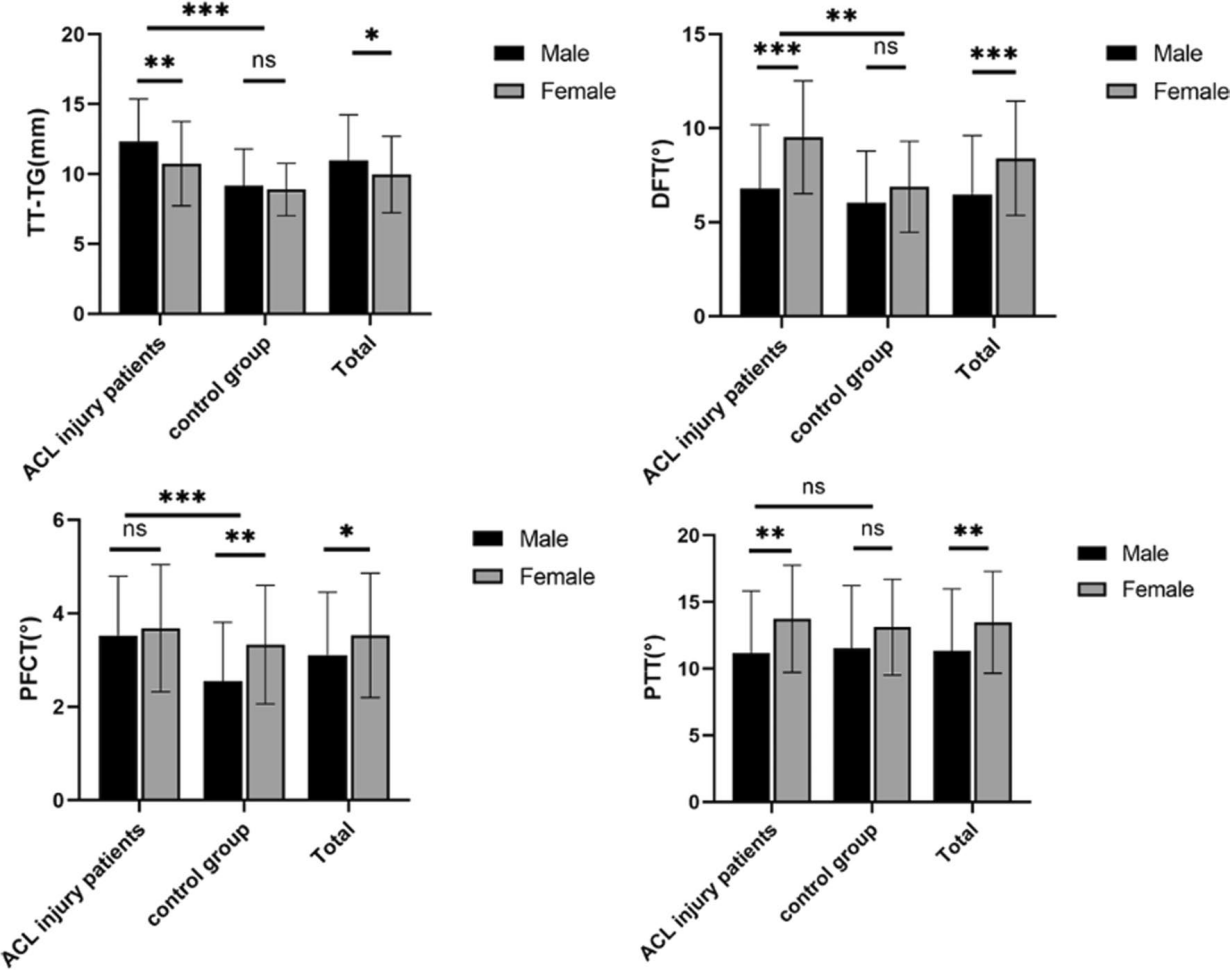
Differences in TT-TG, DFT, PFCT and PTT between study and control groups in different genders. ns., no significant difference, *p < 0.05, **p < 0.01, ***p < 0.001. TT-TG, tibial tubercle to trochlear groove. DFT, distal femoral torsion. PFCT, posterior femoral condylar torsion. PTT, proximal tibial torsion

Table 1 presents the patients’ demographic characteristics. There were no significant differences between all groups regarding age, sex, side of injury or BMI. TT-TG, DFT and PFCT were significantly higher in patients with ACL injury than in controls. However, PTT did not show any significant difference between ACL-injured patients and controls (n.s.). Subgroup analyses also revealed that TT-TG, DFT and PFCT were significantly increased in ACL-injured and ACL + ALL-injured patients compared with controls (*P* < 0.05). Patients in the ACL + ALL injury group had mildly increased TT-TG, DFT and PFCT compared with the ACL injury group, but the differences were not significant (n.s.) (Table 2). In all patients with ACL injury, women had significantly increased DFT and PTT and significantly decreased TT-TG than men. Similarly, in the study population, DFT, PFCT and PTT were significantly increased in women, while TT-TG was significantly lower compared to men (Fig. 4).

**Table 1:** Demographics of the ACL + ALL injury, ACL injury and ACL intact group

	ACL injury group	ACL + ALL injury group	Control group	*P* value
No	63	59	90	
Age* (years)	30.9 ± 7.8	29.7 ± 6.5	30.0 ± 6.7	n.s
BMI* (kg/m^2^)	23.6 ± 2.7	23.9 ± 2.8	23.9 ± 2.7	n.s
Injury site				
Right	33	29	48	n.s
Left	30	30	42	
Gender				
Male	41	40	60	n.s
Female	22	19	30	

*The data are given as the mean and standard deviation

**Table 2:** Distal femoral torsion (DFT) for ACL + ALL injury, ACL injury and ACL intact group measured by S-PCA

Measurement parameter	ACL injury group	ACL + ALL injury group	ACL + ALL injury group and ACL injury group	Control group
TT-TG (mm)	11.6 ± 3.4	11.9 ± 2.7 *(n.s.)	11.8 ± 3.1	9.1 ± 2.4 *(p < 0.001) **#(p < 0.001)** **§(p < 0.001)**
DFT (degree)	7.7 ± 3.5	7.7 ± 3.5 *(n.s.)	7.7 ± 3.5	6.3 ± 2.7 *(p = 0.005) **#(p = 0.009)** **§(p = 0.001)**
PFCT (degree)	3.3 ± 1.3	3.8 ± 1.2 *(n.s.)	3.6 ± 1.3	2.8 ± 1.3 *(p = 0.014) **#(p < 0.001)** **§(p < 0.001)**
PTT (degree)	12.6 ± 4.5	11.4 ± 4.6 *(n.s.)	12.1 ± 4.6	12.1 ± 4.4 *( n.s.) #( n.s.) §(n.s.)

Bold values indicate statistical significance

*Different from ACL injury group; # different from ACL + ALL injury group; § different from ACL + ALL injury group and ACL injury group

*ACL* anterior cruciate ligament, *ALL* anterolateral ligament, *TT-TG* tibial tubercle to trochlear groove distance, *DFT* distal femoral torsion, *PFCT* posterior femoral condylar torsion, *PTT* proximal tibial torsion

**Fig. 5:**
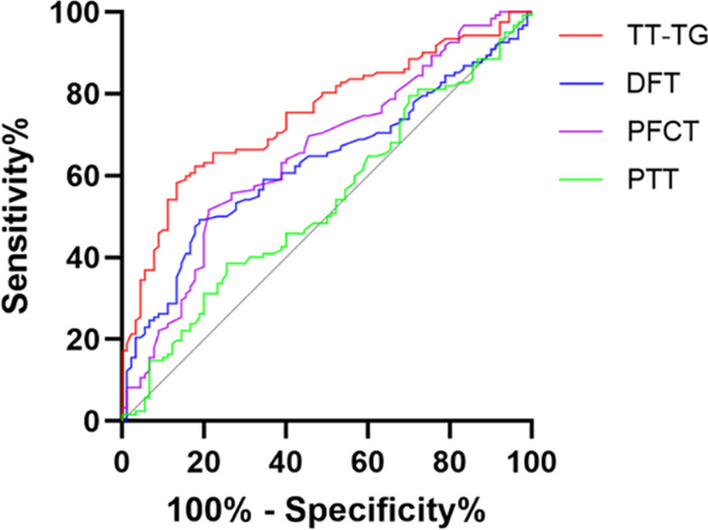
An ROC curve analysis was performed to determine the threshold TT-TG, DFT, PFCT and PTT that were associated with ACL injury

ROC curve analysis showed that in all patients, the AUC value of TT-TG was 0.749 (95% CI 0.684–0.814), and the cut-off value of TT-TG was 11.0 mm (Youden index 0.45), which had a sensitivity of 58% and specificity of 87% for predicting an increased risk of ACL injury (OR 9.05; 95% CI 4.47–18.34). The AUC value of DFT was 0.629 (95% CI 0.554–0.704), and the cut-off value of DFT was 7.9° (Youden index of 0.30), which had a sensitivity of 49% and specificity of 81% for predicting an increased risk of ACL injury (OR 4.16; 95% CI 2.20–7.85). The AUC value of the PFCT was 0.655 (95% CI 0.581–0.729), and the cut-off value of the PFCT was 3.5° (Youden index of 0.31), which had a sensitivity of 52% and specificity of 79% for predicting an increased risk of ACL injury (OR 3.99; 95% CI 2.15–7.40). The AUC value for the PTT was 0.536 and had no predictive value (Fig. 5).

## Discussion

The most important finding of this study was that knee torsional alignment, expressed as TT-TG, was higher in patients with ACL injury than in controls and that it occurred predominantly in the distal femur. No difference in PTT was observed between patients with ACL injuries and controls. It was demonstrated in our study that the primary cause of knee torsion in patients with ACL injury is increased femoral torsion, rather than PTT. However, the relationship between increased knee torsion and combined ALL injury remains unclear.

Knee torsion is a potential axial misalignment which is an anatomical factor leading to altered axial biomechanics, which exhibits differences in various populations as well as in different diseases [17, 24, 25]. For ACL injury, previous biomechanical experiments have shown that for a given quadriceps force, the greater the angle of torsion, the greater the tibial shear, subjecting the ACL to greater stress [26]. Therefore, it is necessary to explore the outliers in patients with non-contact ACL injury and to determine the contributing components of each segment, to provide a theoretical basis for better prevention of non-contact ACL injury.

TT-TG has been previously used to evaluate patellofemoral instability, and subsequent studies have revealed that the TT-TG distance is significantly greater in patients with non-contact ACL injury than in normal knees and correlates with knee torsion, which is considered a similar axial measurement parameter [15, 17, 27]. Usually, a larger TT-TG indicates greater external rotation of the tibia relative to the femur and greater stress on the ACL. The pivot-shift mechanism is the primary cause of non-contact ACL injury, with anterior tibial translation, internal rotation of the tibia and backward movement of the lateral femoral condyle. Therefore, the greater the TT-TG distance, the more internal rotation of the tibia, leading to increased shear forces over the tibia, keeping the ACL under greater tension and increasing the risk of ACL injury. Conversely, when the ACL is injured, the mechanism limiting tibial internal rotation is lost; thus, internal rotation is increased in the tibia relative to the femur, resulting in a decrease in TT-TG [28]. Simultaneously, the ALL acts as a second stabilising mechanism for the ACL, controlling anterolateral rotation with the ACL [29]. The tibia is further internally rotated relative to the femur when the ALL is injured. Our study revealed an increase in TT-TG in ACL injury patients compared to controls; nevertheless, a greater difference in TT-TG may have been observed in patients before ACL injury. Polat et al. revealed an increase in TT-TG in patients with ACL + ALL injury compared with patients with only ACL injury [28]. No differences were observed between ACL injury and combined ALL injury; however, TT-TG before ACL injury may be different.

The correlation between femoral torsion and increased hip anteversion in ACL injuries has been discussed extensively. Alpay et al. revealed that femoral anteversion leading to increased infratrochanteric femoral torsion was a risk factor for non-contact ACL injury [14]. In addition, the distal femoral morphology is associated with an increased risk of ACL injury, with an increased depth of the posterior femoral condyle causing alterations in gait and mechanics between the tibiofemoral joints, leading to ACL injury [9, 30]. Increased internal rotation of the DFT and PFCT, as we noticed, was risk factors for ACL injury. Cadaveric experiments by Bretin et al. revealed that increased internal rotation of the distal femur led to mechanical axis valgus [31]. Valgus of the knee and internal rotation of the femur lead to a pivotal shift and consequent ACL injury. Biomechanical experiments by Omar et al. suggested that internal rotation of the distal femur may lead to a more posterior resting position of the tibia, thereby increasing the magnitude of the pivot shift [32]. These cadaveric and biomechanical experiments provide a theoretical basis for increased distal femoral and posterior condylar torsions.

Previous studies have revealed that internal tibial rotation is a risk factor for ACL injury [33, 34]. When ACL injury occurs, internal rotation of the knee further increases laxity occurrence. However, our results revealed no apparent correlation between PTT and ACL injuries. Winkler et al. discovered that external tibial rotation, an infratubercular deformity occurring predominantly in the distal tibia, was not significantly correlated with PTT [21]. Thus, knee torsion in patients with ACL injury occurs predominantly on the femoral side.

A relationship between increased knee torsion and combined ALL injury was not demonstrated by our findings. TT-TG, DFT and PFCT tended to be higher in patients with ACL + ALL injury than in those with isolated ACL injury; however, the difference was not significant. Further studies on the mechanism of ALL injury are required to clarify the role of knee torsion. The ALL with the ACL theoretically controls anterolateral rotation; thus, increased knee torsion will lead to greater knee rotational stress and aggravate the degree of knee injury [29]. Based on our findings, the relationship between knee torsion and combined ALL injuries remains unclear.

In the study population, DFT, PFCT and PTT were significantly greater and TT-TG significantly decreased in women compared to men. Similar to previous studies, DFT was significantly higher in women than in men. PFCT was significantly higher in patients with lower extremity valgus than in those with neutral or varus [35]. This finding is also consistent with a higher risk of non-contact ACL injury in women and patients with lower extremity valgus [1]. The mechanisms by which differences in knee morphology contribute to an increased risk of ACL injuries are multifaceted. Our findings suggest that knee torsion associated with ACL injury occurs predominantly in the distal femur rather than the proximal tibia, which contributes to a better understanding of the causes of ACL injury. Future kinematic research should clarify the mechanisms of ACL injury by these factors to provide more precise prevention and treatment.

This study has some limitations. Firstly, not all patients underwent contralateral MRI; therefore, assessing anatomical risk factors in the contralateral healthy knee was impossible. Secondly, several torsional indicators have been measured previously mainly using computed tomography, which may differ from our results. However, MRI is more widely used in ACL injuries and has a higher reliability, making our results more valuable [36]. Finally, because our study was retrospective and may have some selection bias, the correlation between knee torsion and the risk of ACL injury and exploring the underlying mechanisms need to be further validated by prospective studies with large sample sizes in the future. The results of this study allow for the identification of people at high risk for non-contact ACL injury in MRI and help physicians target prevention and treatment strategies.

## Conclusions

Knee torsional alignment is associated with ACL injury, predominantly in the distal femur rather than in the proximal tibia; however, its relationship with combined ALL injuries is unclear. This helps clinicians identify patients at risk of non-contact ACL injury and inform them of prevention strategies.

## Data Availability

The datasets used and/or analysed during the current study are available from the corresponding author on reasonable request.

## Abbreviations

ACL: Anterior cruciate ligament
ALL: Anterolateral ligament
BMI: Body mass index
MRI: Magnetic resonance imaging
TT-TG: Tibial tubercle-trochlear groove
DFT: Distal femoral torsion
PFCT: Posterior femoral condylar torsion
PTT: Proximal tibial torsion
ROC: Receiver operating characteristic
Cis: Confidence intervals
AUC: Area under the curve
OR: Odds ratios
